# Genome Assembly of the Ragweed Leaf Beetle: A Step Forward to Better Predict Rapid Evolution of a Weed Biocontrol Agent to Environmental Novelties

**DOI:** 10.1093/gbe/evaa102

**Published:** 2020-05-19

**Authors:** Sarah Bouchemousse, Laurent Falquet, Heinz Müller-Schärer

**Affiliations:** e1 Department of Biology, University of Fribourg, Switzerland; e2 Swiss Institute of Bioinformatics, Fribourg, Switzerland

**Keywords:** biological control agent, *Ophraella communa*, Chrysomelidae, whole-genome sequence, SMRT-cell sequencing, de novo assembly

## Abstract

Rapid evolution of weed biological control agents (BCAs) to new biotic and abiotic conditions is poorly understood and so far only little considered both in pre-release and post-release studies, despite potential major negative or positive implications for risks of nontargeted attacks or for colonizing yet unsuitable habitats, respectively. Provision of genetic resources, such as assembled and annotated genomes, is essential to assess potential adaptive processes by identifying underlying genetic mechanisms. Here, we provide the first sequenced genome of a phytophagous insect used as a BCA, that is, the leaf beetle *Ophraella communa*, a promising BCA of common ragweed, recently and accidentally introduced into Europe. A total 33.98 Gb of raw DNA sequences, representing ∼43-fold coverage, were obtained using the PacBio SMRT-Cell sequencing approach. Among the five different assemblers tested, the SMARTdenovo assembly displaying the best scores was then corrected with Illumina short reads. A final genome of 774 Mb containing 7,003 scaffolds was obtained. The reliability of the final assembly was then assessed by benchmarking universal single-copy orthologous genes (>96.0% of the 1,658 expected insect genes) and by remapping tests of Illumina short reads (average of 98.6 ± 0.7% without filtering). The number of protein-coding genes of 75,642, representing 82% of the published antennal transcriptome, and the phylogenetic analyses based on 825 orthologous genes placing *O. communa* in the monophyletic group of Chrysomelidae, confirm the relevance of our genome assembly. Overall, the genome provides a valuable resource for studying potential risks and benefits of this BCA facing environmental novelties.

## Introduction

Rapid evolution of weed biological control agents (hereafter: BCAs) to both biotic and abiotic environments is poorly understood and so far only little considered both in pre-release and post-release studies of weed biological control programs (Roderick et al. 2012; Szűcs et al. 2019; Müller-Schärer et al. 2020), nor in predictions of future occurrences under climate change using species distribution models (Sun et al. 2017). This is unfortunate as consequences in term of risks and benefits may potentially be far reaching, such as for nontarget attacks of agricultural crops or native endangered species in case of the evolutionary potential for host range expansion and shifts, or for colonizing so far unsuitable habitats for the BCA that are heavily infested by the target plant invader (Messing and Wright 2006).

Since the emergence of high throughput sequencing methods, population genomic approaches have gained in popularity for studying rapid evolutionary changes in introduced species (Bock et al. 2015; Chown et al. 2015; Dlugosch et al. 2015). This has become possible by the availability of reference genomes of high quality and subsequent bioinformatics processing. For instance, the recently published genome of the Colorado potato beetle, *Leptinotarsa decemlineata*, highlighted a high rate of transposable elements (TEs) compared with other beetle species, high levels of nucleotide diversity and recent expansion of introduced populations that might explain why and how this pest species rapidly evolves on novel host plants, under climate change and/or to overcome management practices (Schoville et al. 2018). Unlike as for introduced pest species, genomic applications used to predict or track rapid evolutionary changes remains uncommon in weed BCAs, and therefore, the availability of genomic resources is almost nonexistent. In the present work, we describe the first genome assembly of a phytophagous insect used as a BCA, that is, the leaf beetle *Ophraella communa* LeSage 1986 (Coleoptera: Chrysomelidae) for the control of common ragweed, *Ambrosia artemisiifolia*.

The North American common ragweed, *A. artemisiifolia* L. (Asteraceae), is one of the most prominent plant invaders worldwide, causing economic losses due to severe impacts on human health resulting from its huge amount of highly allergenic pollen and as an important and hard-to-control agricultural crop weed (Essl et al. 2015; Müller-Schärer et al. 2018). Sharing the same native range as common ragweed, the oligophagous chrysomelid beetle *O. communa* is already successfully used as a BCA in Asia (Kim and Lee 2019), and was first reported for Europe in 2013 in the south of the European Alps. Since its arrival in Northern Italy, local aerial pollen concentrations of *A. artemisiifolia* have significantly dropped by 80% compared with beetle-free areas (Bonini et al. 2015; Mouttet et al. 2018). Combining with its recent spreading, this insect became a promising candidate for long-term management of this notorious plant invader also in Europe (Müller-Schärer et al. 2014, 2018).

However, as oligophagous, the leaf beetle was reported feeding on various plants species belonging to the Heliantheae (Asteraceae) tribe (Futuyma and McCafferty 1990), including *Helianthus annuus* L. (sunflower). Its presence on this important economical crop, on which the leaf beetle can even complete its life cycle, was a reason to reject *O. communa* as BCA for Australia (Palmer and Goeden 1991). The risk of this leaf beetle becoming a serious agricultural pest in case of rapid adaptation to crop species remains, however, unclear (Dernovici et al. 2006; Zhou et al. 2011; Lommen et al. 2017), an uncertainty for which the supply of genomic resources and ensuing population genetic studies will provide a most helpful tool for its clarification.

The present study describes the first genome assembly of *O. communa* using sequencing of long-reads. We used several assemblers to get the best genome assembly and then assessed its quality by checking for bacterial contamination and for the presence of conserved insect representative genes. In addition, we evaluated the reliability of this genome assembly to perform population genomic studies by remapping tests, performed a preliminary annotation of functional proteins, and replace the species in the phylogeny of Polyphaga Emery, 1886 (Coleoptera). So far, mitochondrial sequences were the single genetic resources available (e.g., Song et al. 2018). Recently, the antennal transcriptome of *O. communa* has been sequenced to study the molecular basis of olfaction recognition in this insect (Ma et al. 2019). The *O. communa* genome will be used to track the migration and spread history, and to detect potential rapid selection to both novel biotic and abiotic conditions in introduced ranges using genome scans and subsequent outlier analyses.

## Materials and Methods

### Tissue Sampling, DNA Isolation, and Sequencing

A small population of ∼50 individuals of *O. communa* was collected in May 2018 in the Milano area in Italy (GPS coordinates: 45°34′14.63″N, 8°47′7.66″E) and reared under random mating, in the quarantine facilities at the University of Fribourg (Switzerland). After 9 months, corresponding to ∼6 generations, four alive adults (two males and two females) were selected for genomic DNA (gDNA) isolation and preserved in Silica gel. The entire individuals were first dried at 70 °C during 1 h and then pooled before grinding to increase the gDNA content at the end of the DNA isolation step. We assumed that the individuals selected were more genetically similar compared with the individuals of the original population, although this similarity is not expected to be high after six generations of rearing under random mating. This expectation is dependent to the homozygosity rate in the original population and the reproductive skew in the insect species. Under random-mating, the gain of homozygosity from the original population is expected to be ∼6% after six generations of rearing. However, evidences for assortative mating have been reported in this chrysomelid species under controlled conditions (Zhou et al. 2012), suggesting that the level of homozygosity in our reared population may thus have been reinforced according to the extent of the reproductive skew. A DNeasy Plant Mini Kit (Qiagen, Germany) was used to extract gDNA from the pool according to the manufacturer’s protocol.

Five micrograms of the DNA pool was then used to prepare a single library with the PacBio SMRTbell Express Template Prep kit 2.0 (Pacific Biosciences, Menlo Park, CA) according to the manufacturer’s protocol. The resulting library was size selected on a BluePippin system (Sage Science, Inc., Beverly, MA) for molecules larger than15 kilobases (kb). The final library was then sequenced on a PacBio Sequel II instrument (Pacific Biosciences) using five SMRT cells v3, to reach an expectation of ∼50-fold coverage for a genome size estimated at 600 Mb (Petitpierre et al. 2004).

### Genome Assembly

To obtain the most reliable genome assembly, five different assemblers, commonly used for long-reads sequence data (Jayakumar and Sakakibara 2019), were employed: Canu, Flye, MECAT, SMARTdenovo, and wtdbg (supplementary table 1, Supplementary Material online with references and versions used herein). The best assembly corresponds to the one of SMARTdenovo (see Results and Discussion) for which no corrections was applied during the assemblage proceeding. Three rounds of scaffold polishing were therefore performed to improve the accuracy of the genome assembly: For the two first rounds, we used Arrow from the GenomicConsensus package (https://github.com/pacificbiosciences/genomicconsensus, last accessed May 27, 2020) to correct sequence data into consensus sequences with long reads; for the last polishing, we employed Pilon (Walker et al. 2014) to identify and correct inconsistencies between the genome assembly and a set of Illumina short reads. These 150-bp paired-ends (PE) reads are unpublished data obtained from a low-coverage genome-wide sequencing (∼4-fold of coverage per library) done on a NovaSeq system (Illumina Inc., San Diego, CA). The set, encompassing 98 libraries prepared with a Nextera DNA Library Prep (Illumina Inc.), corresponds to 98 single individuals sampled in 13 sites (from 6 to 8 individuals per population) distributed through the current distribution range of the leaf beetle in Italy.

### Quality Assessment

As entire individuals were used for DNA extraction, bacterial contamination of the genome assembly was first investigated using Kraken v0.10.6 (Wood and Salzberg 2014). The completeness of the genome assembly was then assessed by checking for the presence of conserved representative genes. For this purpose, we used BUSCO v3.0.2 (Simao et al. 2015) to benchmark the scaffolds against 1,658 single-copy orthologs of the Insecta_obd9 database. For comparison, the same analyze was performed on the three other chrysomelid genome currently available in online databases (table 1).

**Table 1:** Summary of Global Statistics of the *Ophraella communa* genome assembly and the three other Chrysomelid Genome Assemblies Available in Online Databases (i.e., GenBank-NCBI, ENA, i5k Workspace, and InsectBase)

Species	Total Sequence Length	Sequencing Technology	Coverage	Number of Scaffolds	Scaffold N50 (bp)	Number of Contigs	Contig N50 (bp)	GC%	Protein Count	% of Transposable Elements	Accession Number	References
*Ophraella communa* (ragweed leaf beetle)	774,411,302	PacBio Sequel II	43.3×	7,003	195,463	–	–	31.9%	75,642	58.2	GCA_902651945	Present study
*Leptinotarsa decemlineata* (Colorado potato beetle)	641,992,784	Illumina HiSeq	52.3×	26,908	139,046	45,556	46,596	35.6%	19,038	16.9	GCA_000500325.2	Schoville et al. (2018)
*Diabrotica virgifera virgifera* (western corn rootworm)	2,418,073,815	Illumina HiSeq	50.0×	87,712	489,108	585,680	6,238	36.5%	28,061	–	GCA_003013835.2	Unpublished
*Callosobruchus maculatus* (cowpea weevil)	1,007,816,681	PacBio Sequel I or II	32.0×	15,778	212,245	–	–	37.7%	21,264	63.7	GCA_900659725.1	Sayadi et al. (2019)

In addition, the reliability of using the genome assembly for population genomic studies was evaluated by checking for mapping quality of short reads (i.e., low-coverage whole-genome data, obtained as described above). For this, we used bwa v0.7.17 (Li and Durbin 2009) to map sequences of 18 samples collected in three populations (with six samples for each), one native (Southbury [USA]: 41°27′19.76″N, 73°14′27.78″W) and two introduced (Wuhan [China]: 30°32′42.03″N, 114°25′14.74″E; and Busto Arsizio [Italy]: 45°35′54.60″N, 8°51′51.76″E). Prior to the mapping, the raw sequences were trimmed using trimmomatic v0.36 (Bolger et al. 2014), and only PE reads, representing ∼97% of the trimmed data, were retained for the following analyses. The mapped reads were then filtered on their mapping quality at Q10 and Q20 values using samtools v1.8 (Li et al. 2009).

### Gene Prediction and Phylogenetic Analysis

The gene prediction was performed with Augustus v3.2.3 (Stanke et al. 2006) using parameters “--gff3 = on --species=tribolium2012.” A preliminary annotation of predicted proteins was then performed using Pannzer2 (Toronen et al. 2018).

In order to estimate the proportion of TEs in the genome assembly, a species-specific repeat library was first built using RepeatModeler v1.0.11 (Smit and Hubley 2008–2015). This repeat library was then used with RepBase (http://www.girinst.org/repbase/, last accessed May 27, 2020) to determine the overall TEs content in the genome assembly using RepeatMasker (Smit et al. 2013–2015).

Ortholog groups were identified with OrthoDB v10 (Kriventseva et al. 2019) by comparing the predicted genes of *O. communa* with the proteomes of five Polyphaga beetles (supplementary table 1, Supplementary Material online). To confirm the phylogenic position of the ragweed leaf beetle, we reconstructed a molecular phylogeny from 825 single-copy orthologous proteins identified among the proteomes of 12 Polyphaga beetles including *O. communa* (supplementary table 1, Supplementary Material online) and the proteome of the domestic silkmoth (*Bombyx mori*, Lepidoptera; accession number GCF_000151625.1), selected as an outgroup for the phylogenetic tree. The 825 single-copy orthologous proteins were retrieved for every species using BUSCO. The protein sequences were first aligned with Muscle v3.8.1551 (Edgar 2004), then trimmed to retain only confidently aligned regions with trimAl v1.4.1 (Capella-Gutierrez et al. 2009) and finally concatenated to form a single alignment of every species with catfasta2phyml v1.1 (https://github.com/nylander/catfasta2phyml). The maximum likelihood phylogeny was then estimated using RAxML v8.2.12 (Stamatakis 2014), with the PROTGAMMAJTT substitution model, setting the domestic silkmoth as the outgroup species and performing 100 bootstrap replicates to obtain nodal support values. The phylogenetic tree in Newick format was depicted using Dendroscope v3.6.3 (Huson and Scornavacca 2012).

## Results and Discussion

### General Characteristics of the Final Assembly

In total, 33.98 Gb of raw data were sequenced with an average length of 14.96 kb per read. Excepted for Canu, which failed during the processing, all assemblers generated a genome assembly summarized in supplementary table 2, Supplementary Material online. The SMARTdenovo assembly, displaying the best scores (i.e., the lowest number of scaffolds, highest average size and N50 values and longest minimum and maximum scaffold sizes), was considered as the most reliable assembly for the following proceedings.

After the polishing procedure (results in supplementary table 3, Supplementary Material online), the final genome assembly of the ragweed leaf beetle encompasses 7,003 scaffolds for a genome size of 774 Mb. This final genome size is slightly larger compared with the previous estimation of 600 Mb (Petitpierre et al. 2004), a difference already reported for the Colorado potato beetle genome (Schoville et al. 2018). Most of the global statistics of the *O. communa* genome, summarized in table 1, are in the middle range of the three other chrysomelid genomes (e.g., scaffold N50, table 1).

### Quality Assessment

The taxonomic classification made by Kraken assigned 94.37% of the 7,003 scaffolds to the Colorado potato beetle genome, 4.71% to bacteria genomes, 0.75% to various genomes, and the remaining 0.17% was unassigned. This strong majority of leaf beetle sequences supports an (almost) absence of bacterial contamination in our genome assembly.

**Fig. 1.**
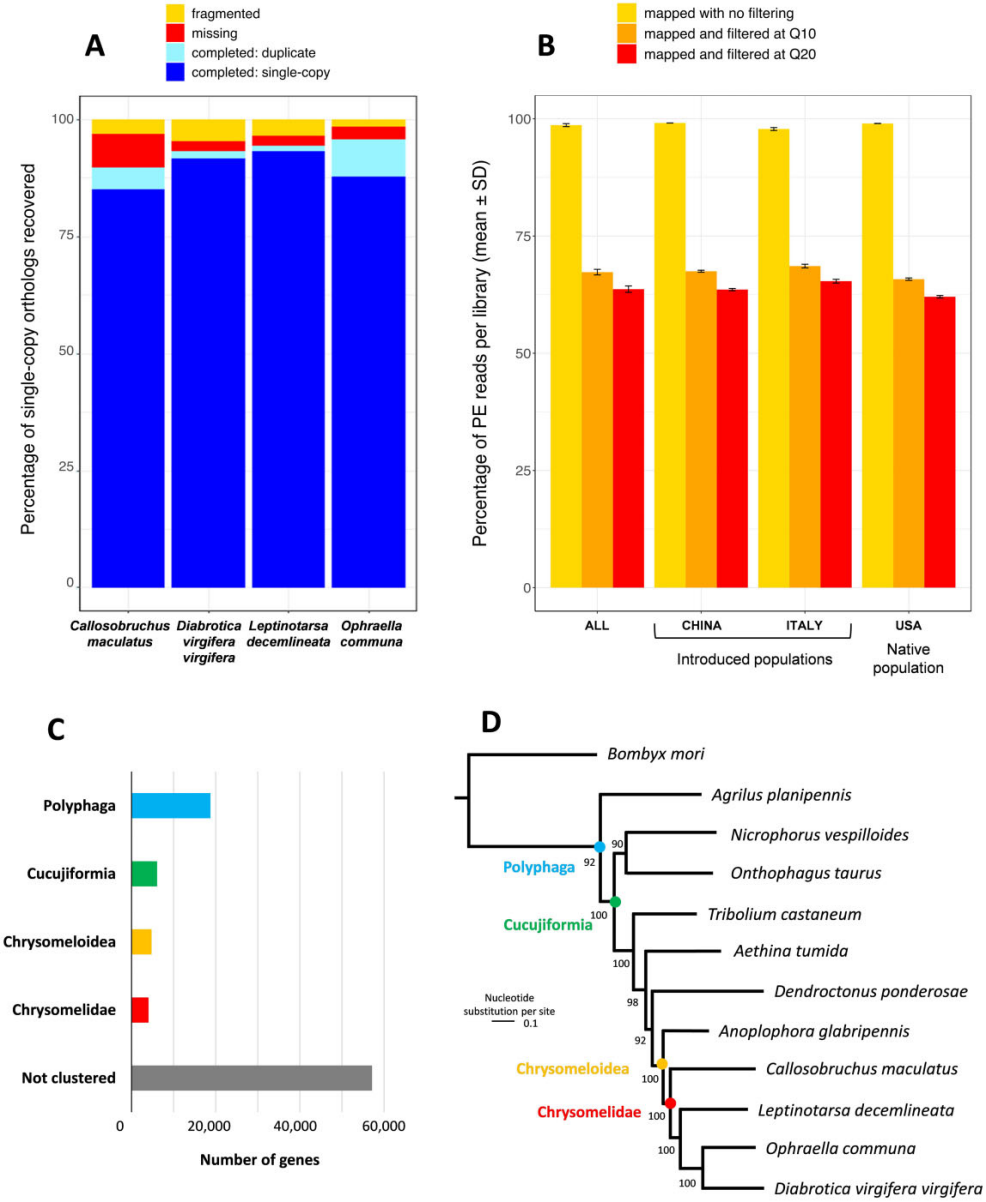
Summary of quality assessments and analysis of the *Ophraella communa* genome assembly: (*A*) Completeness of the present genome assembly and the three Chrysomelid genomes, assessed by the recovery of 1,658 insect benchmarking genes using BUSCO; (*B*) Remapping tests performed on paired-ends (PE) reads after trimming, to assess the reliability of the present genome assembly for population genomic studies of *O. communa*, on all the samples and for each population; (*C*) Taxonomic distribution of the 75,642 genes identified in the proteome of *O. communa* using OrthoDB; (*D*) Maximum-likelihood tree (–ln *L* = –6,269,795.57) built on 825 universal single-copy orthologous proteins among 12 Polyphaga beetles and one lepidoptera (*Bombyx mori*) used as an outgroup, numbers next to nodes indicate internode certainty scores based on 100 bootstrap replicates. The color of dotes at nodes corresponds to the taxonomic color codes in the bar plot (*C*).

The BUSCO assessment indicated an excellent representation of Insect genes, with 96.0% of the 1,658 single-copy orthologs identified as completed (i.e., duplicate and single-copy), whereas only 1.4% and 2.6% were considered as fragmented and missing, respectively, in the assembly. These percentages are similar to the three other chrysomelid genomes (fig. 1*A*).

Disregarding the population level, the remapping tests showed high percentages of mapping (fig. 1*B*), suggesting a high reliability of the genome assembly to map short reads. These percentages, however, dropped down after filtering on mapping quality with averages of 67.3 ± 1.3% and 63.7 ± 1.5% for Q10 and Q20 filters, respectively. This decreasing is explained firstly by the multimapping of reads that generated low values of mapping quality (i.e., average of 28.0 ± 1.1% of PE reads with a mapping quality at 0) and secondly by the stage of fragmentation of the genome that prevented the mapping of reads overlapping with the borders of scaffolds. At the population level, reads of the Italian samples displayed higher mapping qualities (e.g., for Q20, average of 65.6 ± 0.9%) than those of Chinese and North-American samples (e.g., averages for Q20 of 63.0 ± 0.5% and 62.1 ± 0.6%, respectively). Knowing that the genome assembly used DNA extracted from Italian samples, this obvious difference strengthens the reliability of our genome assembly for bioinformatic proceedings.

### Gene Prediction and Phylogenetic Analysis

Overall, 75,642 protein-coding genes were predicted in the genome assembly of *O. communa*. This number represents 82% of the number of unigenes (92,259) predicted by Ma *et al.* (2019) based on antennal transcriptome analysis, which suggests a correct representation of protein-coding genes in our genome assembly. This number is, however, much larger compared with other chrysomelid genomes (table 1), suggesting an overestimation of protein-coding genes probably explained by the large number of TEs in our genome assembly (see results on TEs below). Among the 75,642 protein-coding genes, OrthoDB assigned 24.6% (18,619) in orthologous clusters corresponding to those found in Polyphaga beetles proteomes, and 21.0% of them (3,904) clustered with those found in the Colorado potato beetle proteome (fig. 1*C*).

A high fraction of TEs (58.2%, details per category are provided in supplementary table 4, Supplementary Material online) was identified in our genome assembly. Among them, 68.0% could not be attributed to any repeat sequence categories. This high percentage, already reported for the cowpea weevil genome (Sayadi et al. 2019), suggests a high number of repeat sequences unreported in the database, probably explained by long evolutionary distances to previously known repeats.

The maximum likelihood tree obtained with 825 single-copy orthologous proteins shows that *O. communa* form a monophyletic group with the three other chrysomelid species included in the phylogenetic analysis (fig. 1*D*). The following phylogenetic relationship with the other Polyphaga beetles is congruent with the recent update of evolutionary history of Coleoptera based on 95 protein-coding genes (Zhang et al. 2018).

## Conclusion

We provide the first genome assembly of a phytophagous insect used as a BCA, that is, the leaf beetle *O. communa*, a promising biocontrol candidate against common ragweed, one of the world’s most noxious plant invaders. The various quality assessments showed that the genome assembly described in this study is well suited to be used as a reference genome for *O. communa* and probably also for the other thirteen *Ophraella* species. In addition to the transcriptome recently published, the preliminary annotation carried out on the present genome assembly will supply the first insights into molecular mechanisms involved in rapid evolution. Overall, this new genome provides a valuable resource to determine potential risks and benefits of weed BCA facing environmental novelties.

## Supplementary Material

evaa102_Supplementary_Data
