# Supplementary material for: Genome Assembly of the Ragweed Leaf Beetle: A Step Forward to Better Predict Rapid Evolution of a Weed Biocontrol Agent to Environmental Novelties
Source: Genome Biol Evol. 2020 May 19;12(7):1167–73. doi: 10.1093/gbe/evaa102 (PMC7486951; doi:10.1093/gbe/evaa102)
Supplement: evaa102_Supplementary_Data [file evaa102_supplementary_data.docx]

**Supplementary Table 1.** List of Polyphaga beetles (Coleoptera) used to identify orthologous genes in the proteome of *Ophraella communa* using OrthoDB and to reconstruct the molecular phylogeny using 825 single-copy orthologous proteins.

| Family | Scientific name | Common name | Used for ortholog groups identification | Used for molecular phylogenetic analysis | Accession number |
| --- | --- | --- | --- | --- | --- |
| Buprestidae | *Agrilus planipennis* | emerald ash borer | X | X | GCA_000699045.1 |
| Cerambycidae | *Anoplophora glabripennis* | asian longhorned beetle | X | X | GCA_000390285.2 |
| Chrysomelidae | *Callosobruchus maculatus* | cowpea weevil |  | X | GCA_900659725.1 |
| Chrysomelidae | *Diabrotica virgifera virgifera* | western corn rootworm |  | X | GCA_003013835.2 |
| Chrysomelidae | *Leptinotarsa decemlineata* | colorado potato beetle | X | X | GCA_000500325.1 |
| Chrysomelidae | *Ophraella communa* | ragweed leaf beetle |  | X | GCA_902651945 (this study) |
| Curcullionidae | *Dendroctonus ponderosae* | mountain pine beetle |  | X | GCA_000355655.1 |
| Nitidulidae | *Aethina tumida* | small hive beetle |  | X | GCA_001937115.1 |
| Silphidae | *Nicrophorus vespilloides* | - |  | X | GCA_001412225.1 |
| Scarabaeidae | [*Onthophagus taurus*](https://www.ncbi.nlm.nih.gov/Taxonomy/Browser/wwwtax.cgi?mode=Info&id=166361&lvl=3&lin=f&keep=1&srchmode=1&unlock) | - | X | X | GCA_000648695.1 |
| Tenebrionidae | *Tribolium castaneum* | red flour beetle | X | X | GCA_000002335.3 |

**Supplementary Table 2.** Summary of global statistics of the five assemblers used to compute the genome assembly of *Ophraella communa* using long-reads sequence data. The statistics provided in the table correspond to direct outputs of genome assemblies without any polishing and corrections applied post-assembly.

| Assembler | Pipeline description | Reference | Number of scaffolds | Minimum size (bp) | Median size (bp) | Size average (bp) | Scaffold N50 (bp) | Maximum size (bp) | Total genome size (Mb) |
| --- | --- | --- | --- | --- | --- | --- | --- | --- | --- |
| Canu v1.6 | A complete assembler running in three stages: correction of raw reads, trimming of corrected reads and assemblage of trimmed reads in contigs | Koren et al. 2017 | Failure | | | | | | |
| Flye v2.3.4 | An assembler, running without error correction stage, using repeated approximate sequence matches to find overlaps between reads to generate an initial assembly. Then, it performs an extra repeat classification and analysis step to improve the structural accuracy of the resulting assembly | Kolmogorov et al. 2019 | 29,899 | 1,000 | 40,100 | 57,092 | 96,217 | 2,545,202 | 1 619.0 |
| MECAT v1.0 | A similar assembler than Canu working with four tools: a pairwise mapper of SMRT cells, an SMRT reads reference mapper, a noise corrector and a pipeline for hierarchical assembly | Xiao et al. 2017 | 19,426 | 1,001 | 29,668 | 49,790 | 81,943 | 1,057,697 | 965.4 |
| SMARTdenovo v1.0 | A fast assembler working from all-vs-all raw read alignments without an error correction stage, in five modules: read overlapping, rescue missing overlaps, identify low-quality regions and chimaera and produce better consensus | https://github.com/ruanjue/smartdenovo | 7,003 | 10,854 | 55,013 | 111,317 | 196,124 | 5,115,273 | 779.5 |
| wtdbg v2.3 | A fast assembler for long and noisy reads, working without error correction step, that uses the framework of Bruijn graphs to identify overlapping k-mers among the reads | https://github.com/ruanjue/wtdbg2 | 24,659 | 1,227 | 16,934 | 40,910 | 109,918 | 2,999,164 | 1008.0 |

**References**

Koren S, Walenz BP, Berlin K, Miller JR, Bergman NH, Phillippy AM 2017. Canu: scalable and accurate long-read assembly via adaptive k-mer weighting and repeat separation. Genome Research 27: 722-736. doi: 10.1101/gr.215087.116

Kolmogorov M, Yuan J, Lin Y, Pevzner PA 2019. Assembly of long, error-prone reads using repeat graphs. Nature Biotechnology 37: 540-546. doi: 10.1038/s41587-019-0072-8

Xiao CL, et al. 2017. MECAT: fast mapping, error correction, and de novo assembly for single-molecule sequencing reads. Nature Methods 14: 1072-1074. doi: 10.1038/nmeth.4432

**Supplementary Table 3.** Summary of global statistics of the SMARTdenovo assembly after each round of polishing. The global statistics provided after the 3^rd^ round of polishing correspond to the final assembly and are summarized in the Table 1 of the main text.

| Polishing round | Software | Sequences used  (see details in Material and Methods) | Number of scaffolds | Minimum size (bp) | Median size (bp) | Size average (bp) | Scaffold N50 (bp) | Maximum size (bp) | Total genome size (Mb) |
| --- | --- | --- | --- | --- | --- | --- | --- | --- | --- |
| 1st | Arrow from GenomicConsensus v2.2.2 | Long reads from PacBio Sequel II | 7,003 | 10,801 | 54,412 | 110,720 | 196,121 | 5,110,647 | 775.3 |
| 2nd | Arrow from GenomicConsensus v2.2.2 | Long reads from PacBio Sequel II | 7,003 | 10,809 | 54,430 | 110,676 | 195,698 | 5,111,117 | 775.0 |
| 3rd | Pilon v1.22 | Short paired-ends reads of 150 bp from Illumina NovaSeq | 7,003 | 10,788 | 54,387 | 110,582 | 195,463 | 5,110,064 | 774.4 |

**Supplementary Table 4.** Classification of transposable elements (TEs) in the *Ophraella communa* genome assembly.

| Repeat sequence category | Number of elements | Total length (bp) | Percentage of sequences (%) |
| --- | --- | --- | --- |
| DNA elements | 225,418 | 47,628,566 | 6.15 |
| LINEs | 357,265 | 79,226,053 | 10.23 |
| SINEs | 19,013 | 929,337 | 0.12 |
| LTR elements | 31,961 | 4,724,134 | 0.61 |
| Small RNA | 1,601 | 774,448 | 0.10 |
| Satellites | 6,249 | 2,168,455 | 0.28 |
| Simple repeats | 143,772 | 8,126,832 | 1.05 |
| Low complexity | 39,607 | 697,003 | 0.09 |
| Unclassified | 782,303 | 306,526,606 | 39.58 |
| Total TEs | 1,607,189 | 450,801,434 | 58.21 |
